# Long-Term Weekly Iron-Folic Acid and De-Worming Is Associated with Stabilised Haemoglobin and Increasing Iron Stores in Non-Pregnant Women in Vietnam

**DOI:** 10.1371/journal.pone.0015691

**Published:** 2010-12-30

**Authors:** Gerard J. Casey, Damien Jolley, Tran Q. Phuc, Ta T. Tinh, Dang H. Tho, Antonio Montresor, Beverley-Ann Biggs

**Affiliations:** 1 Department of Medicine (RMH/WH), The University of Melbourne, The Royal Melbourne Hospital, Parkville, Australia; 2 School of Public Health Preventive Medicine, Monash University, The Alfred Centre, Prahran, Australia; 3 National Institute of Malariology, Parasitology and Entomology (NIMPE), Hanoi, Vietnam; 4 Research And Training Centre for Community Development, Hanoi, Vietnam; 5 World Health Organization, Geneva, Switzerland; 6 Centre of Clinical Research Excellence in Infectious Diseases, The Royal Melbourne Hospital, Parkville, Australia; Universidade Federal de Minas Gerais, Brazil

## Abstract

**Background:**

The prevalence of anaemia and iron deficiency in women remains high worldwide. WHO recommends weekly iron-folic acid supplementation where anaemia rates in non-pregnant women of reproductive age are higher than 20%. In 2006, a demonstration project consisting of weekly iron-folic acid supplementation and regular de-worming was set up in two districts in a northern province in Vietnam where anaemia and hookworm rates were 38% and 76% respectively. In 2008 the project was expanded to all districts in the province, targeting some 250,000 women. The objectives of this study were to: 1) examine changes in haemoglobin, iron stores and soil transmitted helminth infection prevalence over three years and 2) assess women's access to and compliance with the intervention.

**Methods and Findings:**

The study was a semi-cross-sectional, semi-longitudinal panel design with a baseline survey, three impact surveys at three-, twelve- and thirty months after commencement of the intervention, and three compliance surveys after ten weeks, eighteen and thirty six months.

**Results:**

After thirty months, mean haemoglobin stabilised at 130.3 g/L, an increase of 8.2 g/L from baseline, and mean serum ferritin rose from 23.9 µg/L to 52 µg/L. Hookworm prevalence fell from 76% to 22% over the same period. After thirty six months, 81% of the target population were receiving supplements and 87% were taking 75% or more of the supplements they received.

**Conclusions:**

Weekly iron-folic acid supplementation and regular de-worming was effective in significantly and sustainably reducing the prevalence of anaemia and soil transmitted helminth infections and high compliance rates were maintained over three years.

## Introduction

Anaemia occurs in as many as 500 million women of reproductive age worldwide, especially in resource-poor countries [1]. Anaemic women feel tired and lethargic and there are adverse consequences for their educational potential as well as reproductive health. Iron deficiency is the primary but not the only cause of anaemia [2]. In pregnancy, iron deficiency and anaemia have been associated with higher maternal morbidity, premature delivery, low birth weight, and iron deficiency and anaemia in infants [3]. A recent study showed improved seven-year survival of children if mothers had taken iron-folic acid supplementation during pregnancy compared to controls and mothers taking other micronutrients [4].

Anaemia rates in pregnant women remain high even though most countries have policies that include recommendations for iron-folic acid supplementation during pregnancy. In Vietnam for example, where antenatal iron supplementation has been recommended since 1993, anaemia rates as high as 62% have been reported in pregnant women [5]. In 2001, the World Health Organization recommended that supplementation to improve iron stores in adolescent girls and women before they enter pregnancy should be considered in areas where anaemia rates are greater than 40% [3]. More recently WHO issued a position statement recommending weekly iron-folic acid supplementation where the prevalence of anaemia in women of reproductive age is greater than 20% [6]. Consideration is now being given to introducing weekly iron-folic acid and regular de-worming programs for adolescent girls and non-pregnant women in high risk areas [7]. With this approach women would potentially take weekly supplements and anthelminthics throughout their reproductive years, and long-term compliance and effectiveness will need to be verified in different settings.

In November 2005 a survey of women of reproductive age (16–45 years) in a northern province of Vietnam identified an anaemia prevalence of 38% and hookworm infection prevalence of 78% [8]. Weekly iron-folic acid supplementation and regular de-worming was introduced in May 2006 in a pilot project. Implementation was initially in two districts and distribution occurred at the village and commune level through established provincial health services. The impact of the intervention on anaemia, iron status and soil transmitted helminth infection at three and twelve months in randomly selected samples has previously been reported [9], [10]. However, data from the baseline cohort of women who also participated in these surveys have not been reported. In 2008 the provincial health authorities assumed full responsibility for program implementation through regular health services, and expanded the intervention province-wide. We now report the impact of and compliance with the intervention after 3 years of implementation in women who took part in the baseline survey and who were still eligible for the program. The objectives of the study were to: 1) examine changes in haemoglobin, iron stores and soil transmitted helminth infection prevalence over time and 2) assess women's access to and compliance with the intervention.

## Materials and Methods

### Ethics Statement

Extensive consultation was undertaken between the project team, communities and community leaders, as well as liaison with village, district and provincial health staff. Village health workers provided participants with information regarding the surveys and written informed consent was documented at the time of enrolment in the surveys. The project was approved by the Human Research Ethics Committee of the Walter and Eliza Hall Institute of Medical Research (Melbourne, Australia, Project No. 03/07); the National Institute of Malariology, Parasitology and Entomology (Hanoi, Vietnam), and Melbourne Health (Melbourne, Australia).

### Study location and population

Yen Bai province, one of 63 provinces in Vietnam, is a mountainous region with a largely rural economy, widespread poverty and diverse ethnic groups. Two districts, Yen Binh and Tran Yen, were chosen for the pilot study. At that time each district had a population of approximately 26,000 women of reproductive age. Weekly iron-folic acid and de-worming tablets were actively distributed through the existing health structure at village and commune level[11].

### Eligibility Criteria for Participants

All women of reproductive age (defined as 15 to 45 years) resident in Yen Binh and Tran Yen districts were eligible to participate in the deworming and weekly iron-folic acid intervention. However, women who became pregnant were advised to switch to daily iron-folic acid supplementation provided at government antenatal clinics. If daily iron was not available, we advised pregnant women to take a double dose of the supplements supplied by the weekly program (ie two tablets weekly). The same eligibility criteria applied when the program was expanded to the whole province. Pregnant women were not provided with de-worming treatment as this is proscribed in pregnancy by the Vietnam Ministry of Health.

### Weekly iron-folic acid and deworming intervention

The intervention began in May 2006 and consisted of one ferrous sulphate/folic acid tablet (200 mg ferrous sulphate equivalent to 60 mg elemental iron/0.4 mg folic acid, UNICEF, Copenhagen) taken weekly, and one albendazole tablet (400 mg, UNICEF, Copenhagen) taken every four months for the first year. At the end of the first year the de-worming schedule was changed to 6-monthly and iron-folic acid tablets were sourced in blister packs from Nam Ha Pharmaceutical Joint Stock Company, a Vietnamese pharmaceutical manufacturer. Before the intervention, two staff of each district Department of Preventive Medicine, two nurses from each commune health station and all village health workers in the two districts (a total of 680 health personnel) were trained about the causes, health risks, treatment and prevention of anaemia and hookworm infection and received promotional and educational materials for the women.

Iron-folic acid and albendazole tablets were distributed from the National Institute of Malariology, Parasitology and Entomology to the provincial implementing agency to the Yen Bai Malaria Control Program office, and through the district Preventive Medicine centres to the Commune Health Stations (for albendazole treatment) and on to the Village Health Workers for distribution of the iron-folic acid supplements to women. All eligible women were encouraged to collect their supplements on a monthly basis from the local village health worker, who also recorded the woman's name and date of distribution and gave advice about possible side-effects and safe storage of supplements. Albendazole tablets were administered as observed treatment on locally designated days either at the commune health station or supervised in the village by a commune health worker. In addition, health workers distributed simple educational materials and conducted or participated in regular community meetings to disseminate more detailed information. The deworming and weekly iron-folic acid intervention was continued in the two districts through 2007, and in 2008 was expanded to all districts in Yen Bai province. The processes undertaken to initiate, promote, maintain and expand the intervention are described elsewhere [10].

### Sample Size

We estimated that a sample size of at least 280 was required in the baseline and follow-up surveys to detect an increase in haemoglobin of 5 g/L with a power of 0.9, a type 1 error of .05, and accounting for clustering with an intraclass correlation of 0.2. This number was also sufficient to detect a reduction in hookworm prevalence of 30%.

### Sampling

We chose four time points for population surveys (baseline, 3, 12 and 30 months).

i) Baseline Survey - November 2005 [8]: The baseline survey was conducted before commencing the intervention and used a stratified multi-stage cluster sampling design. Primary sampling units (villages) were chosen using a ‘probability proportional to size’ random sampling method separately within each district, with half the target sample of villages taken from each district. Secondary sampling units (individual women) were selected randomly from each village using provincial lists.

ii) Anaemia, iron status and hookworm impact surveys - August 2006, May 2007, and October 2008. For the August 2006 and May 2007 surveys we invited baseline participants, as well as a new randomly selected group of eligible women[9], [11]. For the 30 month survey we restricted participation to those women who had participated in the baseline survey. There were some losses to follow-up due to women now being over the eligible age for participation or having moved out of the area.

iii) Compliance Surveys: An independent Vietnamese non-Government Organisation, the Research and Training Centre for Community Development (RTCCD), was commissioned to evaluate efficiency of distribution and women's compliance at ten weeks, seventeen months and three years after commencement of the intervention. For the ten week and seventeen month surveys women were randomly selected from provincial lists of baseline and non-baseline village clusters. For the 3 year survey, women from the original 2005 baseline, ten week and seventeen month surveys were sought together with a random sample of women from another district using probability proportional to size sampling. The data from this third district is not included here. One-to-one interviews were conducted with women and village health workers using structured questionnaires which included questions about their understanding of anaemia, iron deficiency and helminthiasis, distribution of iron-folic acid tablets to the village health workers, the availability of supplements to the women and recall of accessing the de-worming treatment, and taking the weekly iron-folic acid during the previous 10 weeks. Compliance was defined as taking 75% or more of tablets received.

### Samples and testing

Sample collection and laboratory analysis has been previously reported [8] and was the same in each of the surveys. The same teams conducted each survey and consisted of trained phlebotomists, stool preparation and analysis technicians, a demographic recorder/interviewer and a supervisor. Briefly, haemoglobin was assessed at the field site using a HemoCue 201+ (HemoCue AB, Angelholm, Sweden). A 3 mL sample of venous blood was also collected using collection tubes containing a fast clotting agent (samples were centrifuged at the field site and serum was stored on ice for 2–4 hours before freezing to −20°C). Ferritin was measured using a sandwich immunoenzymatic assay (Access® Immunoassay Systems, Beckman Coulter Access Reagents, Fullerton, CA; analytical sensitivity 0.2 µg/L), and soluble transferrin receptor using an enzyme-linked immunoassay (IDeA® sTfR IT, Orion Diagnostica, Espoo, Finland, analytical sensitivity <0.1 mg/L). The ratio of transferrin receptor to log (base 10) ferritin (TfR-F index) was calculated from these results.

Anaemia was defined as a haemoglobin concentration of <120 g/L and iron deficiency as ferritin of <15 µg/L, in accordance with WHO recommendations for women of reproductive age [2]. Transferrin receptor levels above 2.3 mg/L were considered as elevated above the norm, based on the manufacturer's reference interval (0.8–2.3 mg/L). The TfR-F index (TfR/log10 ferritin) implies depletion of iron stores once the ratio exceeds 1.8 [12]. Laboratory analyses were conducted at South Eastern Area Laboratory Services, Sydney, Australia (now South Eastern Sydney and Illawarra Area Health Service).

Folate, vitamin A and vitamin B12 levels were also analysed in subsets of the samples. Folate concentrations were determined by a microtiter technique, exactly as described by O'Broin and Kelleher [13], with chloramphenicol-resistant *Lactobacillus casei* as the test microorganism. Vitamin B12 was measured with the Advia Centaur vitamin B12 assay, a competitive immunoassay based on direct chemiluminescent technology. Vitamin A as serum retinol was measured using high-pressure liquid chromatography [14]. Folate deficiency was defined as less than 10 nmol/L, vitamin A deficiency as a peak less than 1.06 µM and vitamin B12 as less than 150 pmol/L. The analyses were conducted at the University of Otago, Dunedin, New Zealand. The sample size for the B12 estimations was limited by availability of serum in the 12 month survey samples.

Faecal samples were examined for soil transmitted helminth eggs at the field site using standard Kato-Katz methodology [15]. Two slides from each woman were prepared and examined within 30 minutes of sample preparation. Classification of hookworm infection was based on WHO guidelines of 0 eggs per gram (epg) as no infection, >0 and <2000 epg as mild infection and ≥2000 as moderate or severe infection. Soil transmitted helminth infection was categorized as positive if eggs of hookworm (*Ancylostoma duodenale* and *Necator americanus*), *Ascaris lumbricoides* or *Tricuris trichiura* were detected in the stool sample.

### Data entry and checking

Haemoglobin and helminth egg counts were entered into an Excel (Microsoft Office 2003) spreadsheet at the field site. Team supervisors crosschecked entries each day. Demographic data were entered at the end of each survey and laboratory analysis results were entered when they became available.

### Statistical analysis

The results of the baseline survey [8] and the randomly selected women from impact surveys 2006 and 2007 [9] have been previously reported however all data from each survey have now been pooled to improve the precision of the analysis. This semi-cross-sectional, semi-longitudinal design, known as a panel design, is recommended in standard sampling textbooks [16], [17], [18] as an efficient design for the estimation of both change over time and current values of population averages. Recent advances in statistical modelling methods permit analysis of data from panel designs, where individuals have been measured at varying times and at varying numbers of times over the course of the study [19]. Estimates of effects, in the form of changes in population averages over time, are obtained in a way that has been corrected for the correlation between samples from the same individuals at different times and between individuals from the same village.

Primary outcomes were changes in mean haemoglobin levels and hookworm infection prevalence, and secondary outcomes were mean ferritin and prevalence of anaemia and iron deficiency. Haemoglobin values were approximately normally distributed. Ferritin and soluble transferrin receptor values were right-skewed and so log-transformed for analysis. Due to the habit of working outdoors in bare feet, a binary classification of indoor/outdoor work was used as a proxy for exposure risk to soil transmitted helminth infection. Non-Kinh ethnic groups were collapsed into a single category, and education was classified as less than 6 years schooling, 6 to 9 years and more than nine years as these follow the Vietnamese education system.

We present summary results for outcome variables in two ways. First, we display cross-sectional means and confidence intervals, but because these are not independent and so cannot be compared between surveys, we show valid comparative means, prevalences and their confidence intervals.

Data were assembled as longitudinal survey panel data for analysis. For continuous variables the means at each time point were calculated; arithmetic means for haemoglobin and geometric means for the log transformed variables (ferritin, soluble transferrin receptor and the TfR/F-Index). Differences in binary prevalence variables were analysed as the odds ratio between post-implementation and baseline. The odds ratios measure the relative change in risk of disease compared with baseline. Hence an odds ratio  = 0.2 at three months means the risk has dropped to about 20% of baseline. Change over time was analysed using a multilevel mixed-effects model for both linear and logistic regressions. Villages and individuals were incorporated as clusters in the analyses models. Statistical analysis was performed using Stata Revision 11 (StataCorp, 2009, College Station, Texas).

## Results

**Figure 1:**
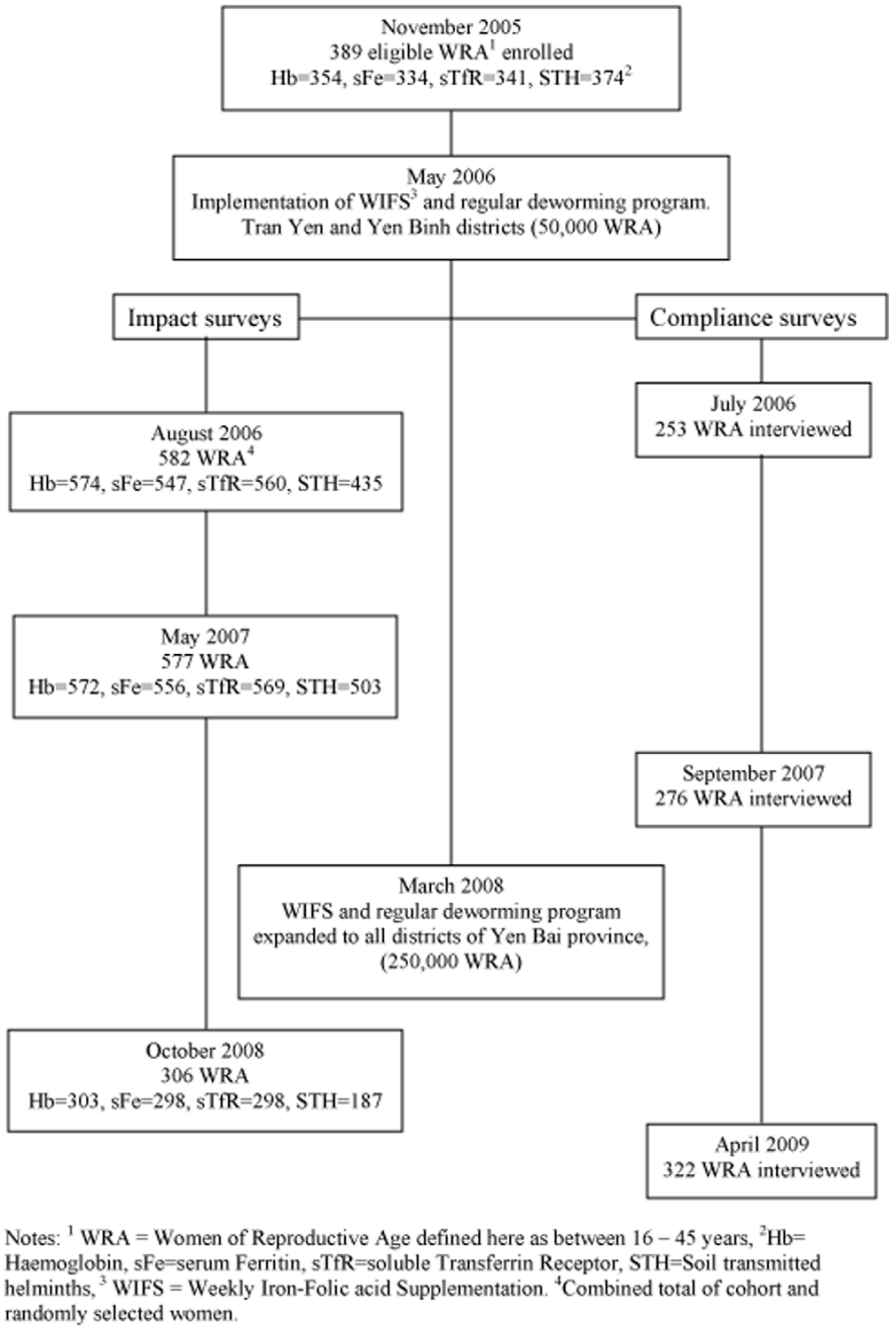
Flow chart of implementation and evaluation of weekly iron-folic acid supplementation and regular de-worming intervention with number of samples analysed from each survey.

**Figure 2:**
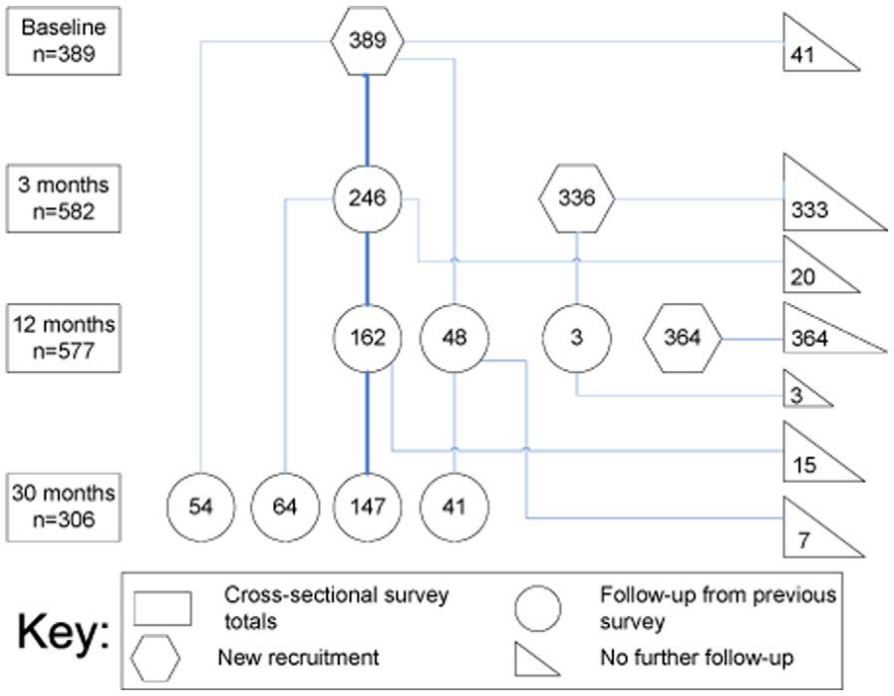
Flow chart of newly-enrolled and follow-up participants.

The sequence, participation rates and sample analysis numbers for the baseline and impact surveys and compliance surveys, and timing of the intervention and expansion, are shown in Figure 1. A total of 1089 women participated in at least one impact survey. The flow of new enrolments and follow up participants is shown in Figure 2. The rectangular boxes at the left indicate the total number of women enrolled in each survey, the hexagons show the number of new enrolments at each survey, the circles show the number of women followed up from a previous survey and the triangles at the right show the number of women from each group who were not part of any subsequent survey.

The demographic data for the participants of the baseline and follow up surveys are shown in Table 1. More than 20% of women had no more than a primary school education and around 80% had not progressed beyond middle high school. At each survey more than 85% of women worked at some form of agricultural activity. There were some variations from the baseline characteristics in the follow up. The increases in age, number of married women and number of children at 30 months were a result of the longitudinal nature of the study. Ethnic minority groups accounted for approximately one third of participants. There was a significant decrease in the amount of meat consumed at the 30 month survey compared to baseline (p<0.001).

**Table 1:** Comparative demographic and socio-economic status of women participating in impact surveys at each time point; statistical test results are not shown, since such tests require mixed model analysis.

	Baseline^1^		3 months		12 months		30 months	
Demographic/socioeconomic factor	Total n	Mean ± SD or Frequency (%)	Total n	Mean ± SD or Frequency (%)	Total n	Mean ± SD or Frequency (%)	Total n	Mean ± SD or Frequency (%)
Age	389	30.1±7.9	580	32.0±8.2	577	33.3±7.8	306	33.9±7.7
Marital status	389		575		526		306	
Unmarried		54 (14%)		76 (13%)		38 (7%)		17 (6%)
Married		332 (85%)		494 (86%)		483 (92%)		283 (92%)
Widowed/divorced		3 (1%)		5 (1%)		5 (1%)		6 (2%)
Pregnant	389	7 (2%)	574	16 (3%)	523	21 (4%)	306	5 (2%)
No. of children	389	1.7±1.1	575	1.8±1.1	524	1.9±1.0	306	2.0±.9
Meat consumption	375	4.1±2.9	565	3.7±2.2	463	3.8±2.4	302	3.2±2.3
Education	385		573		508		306	
none/primary (1–5)		86 (22%)		160 (28%)		128 (25%)		83 (27%)
6–9 years		215 (56%)		297 (52%)		288 (57%)		159 (52%)
>9 years		84 (22%)		116 (20%)		92 (18%)		64 (21%)
STH infection risk (work)	385		574		507*		306	
Office, housewife,other (low)		55 (14%)		82 (14%)		29 (6%)		33 (11%)
Outdoors (high)		330 (86%)		492 (86%)		478 (94%)		273 (89%)
Ethnicity	388		575		530		305	
Kinh		258 (66%)		324 (56%)		352 (66%)		194 (64%)
Other		130 (33%)		251 (44%)		178 (34%)		111 (36%)
Place of residence	389		582		577		306	
Tran Yen		224 (58%)		268 (46%)		291 (50%)		168 (55%)
Yen Binh		165 (42%)		314 (54%)		286 (50%)		138 (45%)

1 Represents baseline survey conducted in November 2005 prior to implementation of WIFS and de-worming in May 2006.

The cross-sectional means or prevalences of the primary and secondary outcomes are shown in Table 2.There was no evidence of any impact of pregnancy on the change in means (data not shown) and so pregnant women (< = 4% of sample population in each survey) were included in the analysis. There were consistent increases in haemoglobin between baseline and three- and twelve months post-intervention with the sample mean stabilised around 130 g/L at the 30 month survey. Ferritin levels rose consistently at all time points from the baseline to the final survey. Iron status indicators and soil transmitted helminth prevalence all improved over time. The prevalence of depleted iron stores, as measured by the TfR/Fe-Index, dropped by 57% from 67/331 (20%, 95% CI [14%, 26%]) to 26/296 (9%, 95% CI [4%, 13%] OR 0.22, 95% CI [0.09, 0.53] at 30 months.

**Table 2:** Means and prevalences over time for surveyed health indicators; statistical test results can be inferred from confidence intervals.

Outcome	Survey	N of	Mean	95% CI	
	(months)	values			
IRON MEASURES					
Haemoglobin	Baseline	354	122.1	119.8	124.4
(g/L)	3	574	126.4	125.0	127.8
	12	572	131.0	128.8	133.2
	30	303	130.3	128.3	132.3
Serum ferritin *	Baseline	334	28.1	24.1	32.7
(µg/L)	3	547	38.4	35.1	41.9
	12	556	44.7	41.1	48.6
	30	298	52.0	44.9	60.2
Soluble Tranferrin Receptor * (mg/L)	Baseline	341	1.7	1.6	1.8
	3	560	1.7	1.6	1.7
	12	569	1.4	1.3	1.5
	30	298	1.4	1.3	1.5
IRON STATUS					
Anaemia	Baseline	354	38%	31%	45%
	3	574	27%	22%	32%
	12	572	20%	16%	24%
	30	303	19%	14%	24%
Iron deficiency	Baseline	334	19%	13%	25%
	3	547	8%	6%	10%
	12	556	6%	4%	8%
	30	298	6%	3%	9%
Iron-deficiency anaemia	Baseline	334	14%	10%	19%
	3	546	6%	4%	8%
	12	555	4%	2%	6%
	30	297	4%	1%	6%
INFECTIONS					
Hookworm infection	Baseline	374	76%	68%	84%
	3	435	57%	50%	63%
	12	503	26%	21%	31%
	30	187	22%	12%	32%
*A. lumbricoides*	Baseline	374	20%	13%	26%
	3	435	6%	3%	9%
	12	503	4%	2%	6%
	30	187	4%	1%	7%
*T. trichiura*	Baseline	374	29%	24%	35%
	3	435	19%	14%	25%
	12	503	11%	7%	14%
	30	187	10%	4%	15%

*Geometric means.

**Figure 3:**
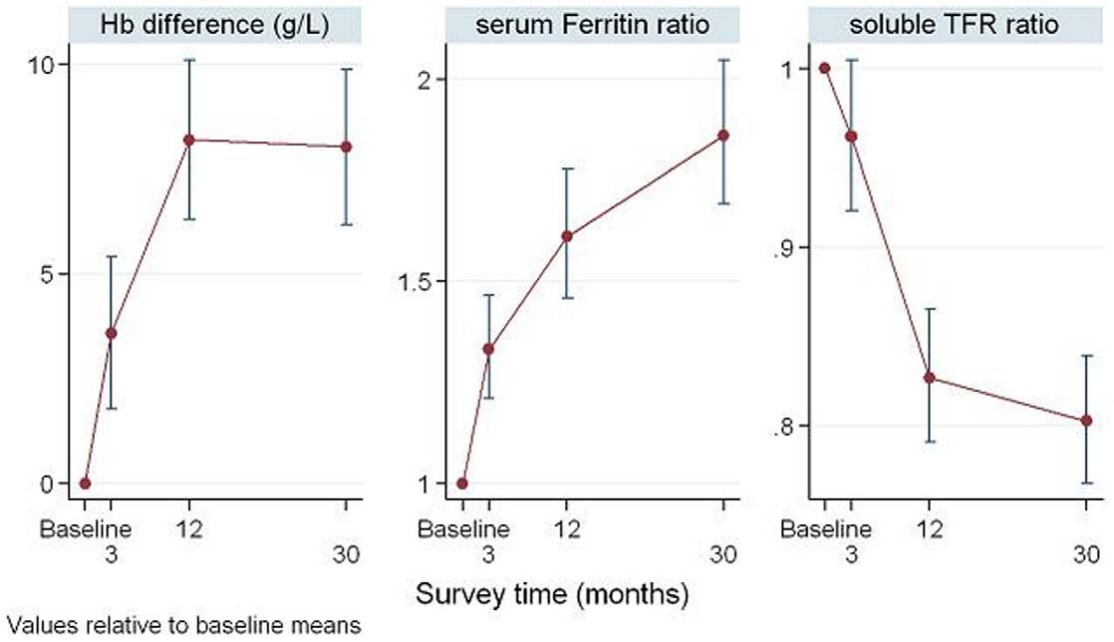
Estimated absolute changes in haemoglobin from baseline and the ratios of follow-up serum ferritin and soluble transferrin receptor relative to baseline; 95% confidence intervals are shown as vertical bars around each estimate and were computed using linear mixed models to take account of study design. # This figure shows the results of the mixed-effects model and non-overlapping confidence intervals indicate statistical significance.

Comparisons in outcomes between surveys can be seen in Figures 3 to [Fig pone-0015691-g004] 5. The absolute change in haemoglobin and the ratio of follow up ferritin and soluble transferrin receptor to baseline are shown in Figure 3. There was an 84% increase in mean ferritin level and a 30% decrease in mean soluble transferrin receptor level from baseline to 30 months.

**Figure 4 pone-0015691-g004:**
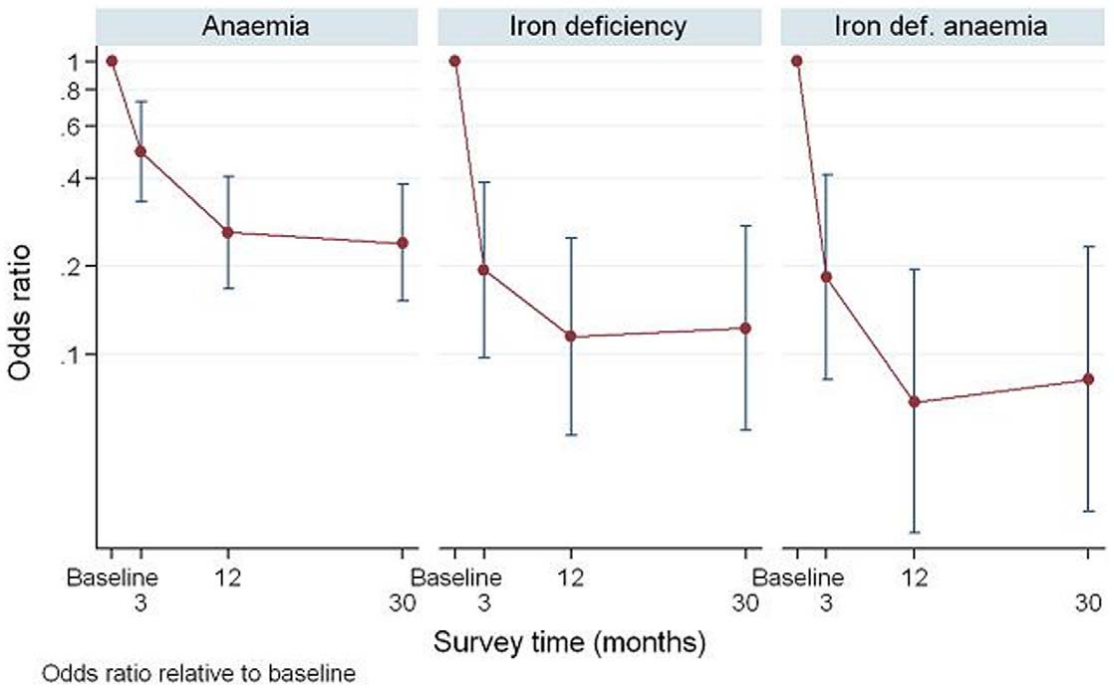
Estimated odds ratios for anaemia, iron deficiency and iron deficiency anaemia relative to baseline; 95% confidence intervals are shown as vertical bars around each odds ratio estimate, and were computed using logistic mixed models to take account of study design. # This figure shows the results of the mixed-effects model and non-overlapping confidence intervals indicate statistical significance.

**Figure 5:**
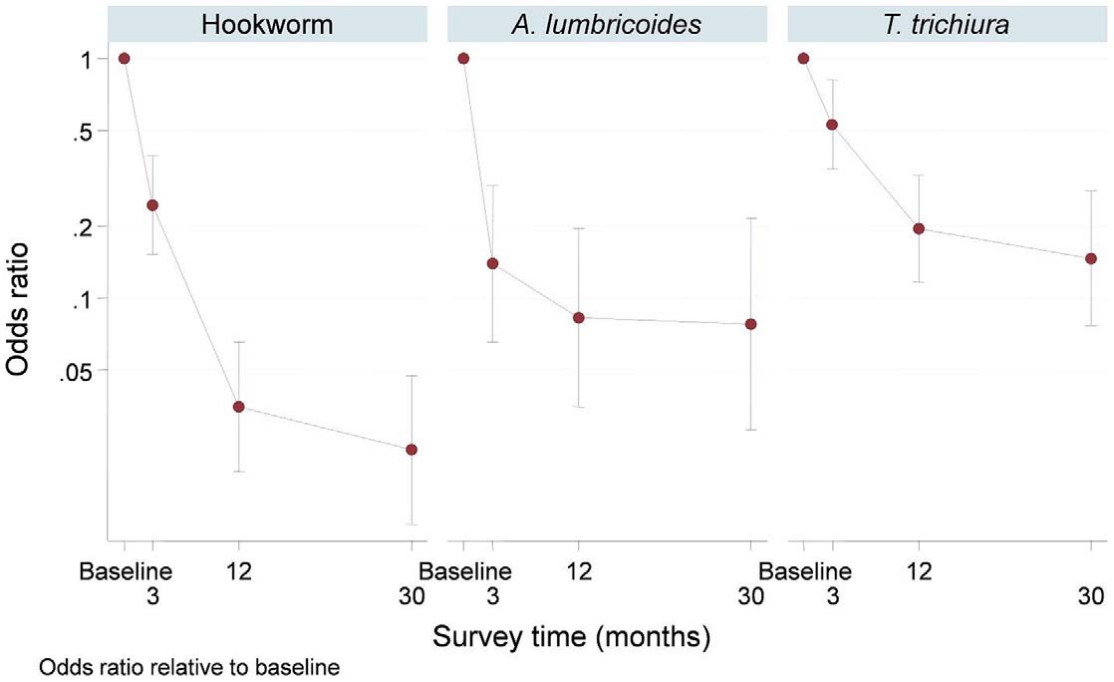
Estimated odds ratios for each species of soil transmitted helminth relative to baseline; 95% confidence intervals are shown using vertical bars and were computed using logistic mixed models to take account of study design. # This figure shows the results of the mixed-effects model and non-overlapping confidence intervals indicate statistical significance.

Univariable analysis of the change in ferritin over time identified the presence of hookworm infection as a potential confounder and so this was incorporated into the regression model for analysis of anaemia and iron status. The odds ratios for anaemia, iron deficiency and iron deficiency anaemia are shown in Figure 4. Compared to baseline the risk of anaemia, iron deficiency and iron deficient anaemia at thirty months was approximately 24%, 12% and 8% respectively. Of the residual anaemia after 30 months, 46/57 (81%) was not associated with iron deficiency. Half of those with residual anaemia were of non-Kinh ethnicity, although non-Kinh women made up only 36.4% of the total survey population.

The odds ratios for each species of soil transmitted helminth relative to baseline are shown in Figure 5. At thirty months the risk of hookworm, *A. lumbricoides* and *T. trichiura* infection compared to baseline was approximately 2%, 8% and 15% respectively.

To assess whether the concentration of folic acid of 400 µg in a standard iron-folic acid tablet was sufficient to alter women's folate levels, folate was assessed in 310/354 (88%) baseline samples and 459/572 (80%) 12 month survey samples. There was insufficient remaining from the other samples for analysis. At baseline the sample mean folate was 25.9 nmol/L with a non significant change to 25.1 nmol/L at 12 months. Folate sufficiency was observed in 291/310 women (94%, 95% CI [91%, 97%]) with a non-significant change to 422/459 (92%, 95% CI [89, 94]) at 12 months.

To identify if vitamin A deficiency was a possible contributing factor to baseline anaemia, 319 remaining serum samples were analysed. The mean vitamin A peak was 2 mM and sufficiency was observed in 314/319 (98%, 95% CI [97%, 100%]). Similarly Vitamin B12 was measured in 109/572 (19%) of samples from the 12 month survey to identify if vitamin B12 deficiency was a possible contributing factor to residual anaemia. Of these, 27/109 (25%) were anaemic compared to the anaemia prevalence of 111/572 (19%) in the total sample population. Vitamin B12 sufficiency was observed in 106/109 women (97% (95% CI [97%, 100%]).

Compliance with taking iron-folic acid tablets was dependent on receiving the tablets. Distribution and compliance rates are shown in Table 3. Initially the distribution was reported as almost 100% however over time this reduced to a long-term rate of greater than 80%. In this report compliance is defined as taking at least 75% of tablets received. After an early period of uncertainty about the value of the supplements, compliance remained above 80% among those who received the supplements.

**Table 3:** Proportion of women who reported receiving iron-folic acid tablets at each survey (distribution) and proportion of those who took at least 75% of tablets received (compliance).

Survey	Distribution			Compliance		
	n	Received	95% C I	n	Took 75% or more	95% C I
July 2006	253	99%	[98% 100%]	249	51%	[37% 65%]
September 2007	276	88%	[79% 98%]	243	90%	[84% 95%]
April 2009	322	81%	[68% 94%]	261	87%	[80% 93%]

## Discussion

We report the impact of a long-term, universal, free weekly iron-folic acid supplementation and de-worming program on levels of haemoglobin and serum ferritin, and the prevalence of anaemia, iron deficiency, iron deficiency anaemia and soil transmitted helminth infections in a population of rural women of reproductive age in north-western Vietnam. These results demonstrate that Vietnamese women with reliable ongoing access to this weekly program maintained high compliance, had a sustained reduction in anaemia rates, stabilised haemoglobin concentrations and showed continued improvement in iron stores and reduction in hookworm infection prevalence rates during the follow-up period.

Evaluation by an independent agency found that after three years of the intervention, more than 80% of women were regularly receiving supplements and more than 85% of these were taking 75% or more of supplements received. This suggests that women remained strongly committed to the intervention, and that distribution mechanisms were efficient.

Women reported that the iron tablets made them feel better after two or three months and that this was a factor in their decision to continue taking the tablets (Dr. Luca Tommaso Cavalli-Sforza, WHO Mission Report, MR/2010/0126, unpublished).

There are few published studies of long term iron-folic acid supplementation programs for non-pregnant women. One study evaluated a weekly iron-folic acid supplementation and de-worming program targeting adolescent girls in Uttar Pradesh, India over four years [20]. Hemoglobin levels and compliance were assessed in a random sample of schoolgirls and non-schoolgirls. Results showed improved haemoglobin, reduced anaemia rates, and a compliance rate of over 85% in girls whether or not they attended school. Ferritin levels as a measure of iron stores were not tested. Although supplementation programs are more easily administered through schools, the study showed that distribution to adolescent girls is also possible through community mechanisms.

Our study extends these findings, showing a beneficial response in iron stores over a sustained period when a weekly iron-folic acid supplementation and de-worming program is effectively delivered in a rural setting through village-based health services to all adolescent girls and women of reproductive age. Taken together these studies suggest that effective distribution and promotion systems can be developed for different target groups and settings.

The finding of hookworm as a factor influencing ferritin levels is in keeping with previous reports [21]. These soil transmitted helminths cause significant intestinal blood loss. It has been estimated that even a light hookworm infection can cause the loss of almost 2 ml of blood per gram of faeces [22]. Hookworm infection readily responds to treatment with a single 400 mg dose of albendazole, which needs to be repeated every six months or so in endemic areas where re-infection is common. The program had indirect benefits of reducing the prevalences of *A. lumbricoides* and *T. trichiura,* which are not usually associated with iron deficiency anaemia in adults. While *T. trichiura* does not respond as readily to a single albendazole treatment, the results presented here show that regular treatment has a cumulative impact on infection prevalence. We considered it unethical to conduct separate arms of the intervention to assess the relative contributions of de-worming and iron supplementation to improved iron status as there is strong evidence supporting the effectiveness of these interventions when given separately, and the relative contribution of each will vary depending on dietary iron intake, and helminth prevalence in the population. The findings presented here suggest that iron-folic acid supplementation programs for non-pregnant women are best combined with regular de-worming to achieve maximal benefit in areas where both constitute a public health problem.

The observation that residual anaemia was present in 19% of participants at the end of the study is similar to that observed previously in Vietnam [23], where 18% of non-pregnant participants had anaemia not associated with iron deficiency after 12 months of weekly iron-folic acid supplementation. This residual is unlikely to be explained by folate, vitamin B12 or vitamin A deficiency as testing in our study showed that these deficiencies were uncommon in this population. A possible explanation is that anaemia was caused by a genetic disorder such as thalassemia or other hemoglobinopathy, which are common in SE Asian populations [24]. This is supported by our finding that ethnic minority women were disproportionately represented in the final non-iron deficient anaemia group.

The main strength of the study was that it evaluated a long-term weekly iron-folic acid supplementation and de-worming program that was integrated into the primary health system and implemented by local health staff, and so represents a realistic estimate of what can be achieved in a poor rural community when an intervention is developed in consultation with all levels of the community and health services, distribution is reliable and supplements are provided free of charge. The random selection of an adequate sample, the determination of iron status, the high compliance as verified by independent monitors, and the long duration of follow-up were all important elements of the program evaluation.

The main limitation of the study was the absence of a control group to ascertain that the findings observed were due to the intervention and not unrelated factors. However, it was felt to be unethical and unnecessary to impose an open-ended restriction on women taking iron tablets in order to fulfill the requirements of a control group given the known health benefits of iron. Therefore confounding factors may have influenced the results. One possible confounder was meat intake, which if it increased might have explained the observed increase in ferritin levels and decrease in iron deficiency anaemia. We questioned participants about meat intake at each survey and the inclusion of meat as a regularly consumed food in the ferritin model showed no confounding effect. The latter part of the study coincided with unstable economic conditions globally and so it is unlikely that the improvements in iron status were due to improvements in the general socioeconomic circumstances of the women who participated in the study. This is supported by the decreased meat consumption we observed in the last survey.

Another limitation was the low return of stool samples for analysis at the 30 month survey but the direction and effect of possible bias remains open to speculation.

We conclude that a weekly iron-folic acid supplementation and regular de-worming program was effective in significantly and sustainably reducing the prevalence of anaemia and soil transmitted helminth infections and improving iron stores in non-pregnant women over a three-year period. High compliance rates were maintained and the population mean of haemoglobin increased by 8 g/L and ferritin by 24.4 µg/L. These results provide compelling evidence for national policy makers and other stakeholders to consider this approach in areas with high rates of anemia in women of reproductive age.

## References

[1] McLean E, Cogswell M, Egli I, Wojdyla D, de Benoist B (2009). Worldwide prevalence of anaemia, WHO Vitamin and Mineral Nutrition Information System, 1993-2005.. Public Health Nutr.

[2] WHO/CDC (2005). Assessing the iron status of populations: Report of a joint World Health Organization/Centers for Disease Control and Prevention technical consultation on the assessment of iron status at the population level..

[3] WHO (2001). Iron deficiency anaemia - assessment, prevention and control. A Guide for Programme Managers. Geneva: World Health Organization.. WHO/NHD/01.3.

[4] Christian P, Stewart CP, LeClerq SC, Wu L, Katz J (2009). Antenatal and postnatal iron supplementation and childhood mortality in rural Nepal: a prospective follow-up in a randomized, controlled community trial.. Am J Epidemiol.

[5] Trinh LT, Dibley M (2007). Anaemia in pregnant, postpartum and non pregnant women in Lak district, Daklak province of Vietnam.. Asia Pac J Clin Nutr.

[6] WHO (2009). Weekly iron-folic acid supplementation (WIFS) in women of reproductive age: its role in promoting optimal maternal and child health. Position statement. Geneva: World Health Organization.. http://www.searo.who.int/LinkFiles/Nutrition_for_Health_and_Development_WHO_weekly_viron_folic_acid.pdf.

[7] Viteri FE, Berger J (2005). Importance of pre-pregnancy and pregnancy iron status: can long-term weekly preventive iron and folic acid supplementation achieve desirable and safe status?. Nutr Rev.

[8] Pasricha SR, Caruana SR, Phuc TQ, Casey GJ, Jolley D (2008). Anemia, iron deficiency, meat consumption, and hookworm infection in women of reproductive age in northwest Vietnam.. Am J Trop Med Hyg.

[9] Casey G, Phuc T, MacGregor L, Montresor A, Mihrshahi S (2009). A free weekly iron-folic acid supplementation and regular deworming program is associated with improved hemoglobin and iron status indicators in Vietnamese women.. BMC Public Health.

[10] Mihrshahi S, Casey GJ, Montresor A, Phuc TQ, Thach DT (2009). The effectiveness of 4 monthly albendazole treatment in the reduction of soil-transmitted helminth infections in women of reproductive age in Viet Nam.. Int J Parasitol.

[11] Phuc T, Mihrshahi S, Casey G, Phu L, Tien N (2009). Lessons learned from implementation of a demonstration program to reduce the burden of anemia and hookworm in women in Yen Bai Province, Viet Nam.. BMC Public Health.

[12] Suominen P, Punnonen K, Rajamaki A, Irjala K (1998). Serum transferrin receptor and transferrin receptor-ferritin index identify healthy subjects with subclinical iron deficits.. Blood.

[13] O'Broin S, Kelleher B (1992). Microbiological assay on microtitre plates of folate in serum and red cells.. J Clin Pathol.

[14] Thurnham DI, Smith E, Flora PS (1988). Concurrent liquid-chromatographic assay of retinol, alpha-tocopherol, beta-carotene, alpha-carotene, lycopene, and beta-cryptoxanthin in plasma, with tocopherol acetate as internal standard.. Clin Chem.

[15] Ash LR, Orihel TC, Savioli L, Sin MA, Montresor A (2004). Training manual on diagnosis of intestinal parasites - tutor's guide. Geneva: World Health Organization.. http://www.who.int/publications/en/.

[16] Cochran WG (1977). Sampling Techniques..

[17] Kasprzyk D, Duncan GJ, Kalton G, Singh MP (1989). Panel Surveys..

[18] Kish L Survey Sampling..

[19] Rabe-Hesketh S, Skrondal A (2005). Mulilevel and longitudinal modelling using Stata..

[20] Vir SC, Singh N, Nigam AK, Jain R (2008). Weekly iron and folic acid supplementation with counseling reduces anemia in adolescent girls: A large-scale effectiveness study in Uttar Pradesh, India.. Food Nutr Bull.

[21] Stoltzfus RJ, Dreyfuss ML, Chwaya HM, Albonico M (1997). Hookworm control as a strategy to prevent iron deficiency.. Nutr Rev.

[22] WHO (1999). Monitoring helminth control programmes. Geneva: World Health Organization.. WHO/CDS/CPC/SIP/99.3.

[23] Berger J, Thanh HT, Cavalli-Sforza T, Smitasiri S, Khan NC (2005). Community mobilization and social marketing to promote weekly iron-folic acid supplementation in women of reproductive age in Vietnam: impact on anemia and iron status.. Nutr Rev.

[24] WHO (2006). Thalassemia and other hemoglobinopathies: Report by the Secretariat.. http://apps.who.int/gb/ebwha/pdf_files/EB118/B118_5-en.pdf.

